# The relationship between symptoms of adenomyosis and current ultrasonographic diagnostic criteria

**DOI:** 10.3389/fendo.2026.1924781

**Published:** 2026-10-07

**Authors:** Sofia Ivanisevic, E. Shirin Dason, Christina Hanna, Emma Rebecca Evelyn Sparks, Stella Wang, Ella Huszti, Rebecca Ngu, Alexander Hartman, Mara Sobel

**Affiliations:** 1 Temerty Faculty of Medicine, University of Toronto, Toronto, ON, Canada; 2 Department of Obstetrics and Gynaecology, University of Toronto, Temerty Faculty of Medicine, Toronto, ON, Canada; 3 Biostatistics Department, University Health Network, Toronto, ON, Canada; 4 Mount Sinai Hospital, Toronto, ON, Canada; 5 True North Imaging, Toronto, ON, Canada

**Keywords:** adenomyosis, diagnostic criteria, dysmenorrhea, heavy menstrual bleeding, infertility, transvaginal ultrasound

## Abstract

**Introduction:**

Adenomyosis is a uterine disorder defined by presence of endometrial glands and stroma within the myometrium with surrounding myometrial hyperplasia. Research has shown that ultrasound (US) can play a key role in early adenomyosis diagnosis and treatment. The Morphological Uterus Sonographic Assessment (MUSA) recently published standardized criteria for ultrasonographic diagnosis of adenomyosis, but the relationship between these criteria and clinical symptoms, has yet to be clinically validated.

**Objectives:**

To assess the prevalence of MUSA ultrasound criteria of adenomyosis and relationship between rates of ultrasound features and clinical symptoms of adenomyosis.

**Methods:**

This is an analytical cross-sectional study of patients aged 18–52 receiving a transvaginal ultrasound for any indication at a single outpatient imaging center. Patients were excluded if they had a hysterectomy and/or bilateral oophorectomy, were in menopause, or had active gynecologic malignancy. Patients completed a survey to assess for infertility or fertility-related healthcare use, heavy menstrual bleeding, and pelvic pain. Ultrasound images were assessed by a radiologist using MUSA criteria to evaluate for adenomyosis. Multivariate analyses assessed association between MUSA features and patient-reported symptoms.

**Results:**

Of 271 recruited participants, 11.4% had at least one direct feature of adenomyosis, 57.6% had no direct features but at least one indirect feature, and 31.0% had no features. After controlling for potential confounders in multivariable analysis, patients with direct features resulted in 5.955-point higher heavy bleeding scores compared to those with no features (95% CI 1.970-9.939, p=0.004). When controlling for covariates, the association between indirect features and adenomyosis symptoms were not significant. Subjects with middle myometrium invasion reported increased rates of infertility (p=0.006), whereas subjects with outer myometrial involvement reported significantly higher heavy bleeding (p=0.002).

**Conclusions:**

Per MUSA criteria, 1 in 10 participants displayed ultrasonographic findings strongly suggestive of presence of adenomyosis. Patients with direct features were more likely to experience heavy menstrual bleeding. Layer of uterine involvement were found to have greater association with infertility and pelvic pain than any one MUSA feature.

## Introduction

Adenomyosis is a disease of the uterus characterized histologically by the presence of endometrial glands and stroma within the myometrial tissue, and clinically by pelvic pain, abnormal uterine bleeding, and infertility (1–4). Currently, transvaginal ultrasound (TVUS) is recommended as a first-line diagnostic imaging modality when adenomyosis is suspected (5, 6), however, up to 30% of women with signs of adenomyosis on ultrasound may be asymptomatic (1). In effort to standardize TVUS diagnosis of adenomyosis, a proposed reporting system was established using the Morphological Uterus Sonographic Assessment (MUSA) terminology for myometrial lesions, grouping findings into direct and indirect features (5, 7). Direct MUSA features of adenomyosis on TVUS are defined as findings of ectopic myometrial tissue, and include myometrial cysts, hyperechogenic islands, and echogenic subendometrial lines and buds (7). Indirect MUSA features on TVUS are defined as secondary findings related to endometrial tissue in the myometrium, and include globular uterus, asymmetrical myometrial thickening, fan-shaped shadowing, irregular or interrupted junctional zone, and translesional vascularity (7). Indirect features may be present secondary to other pathologies, such fibroids, intracavitary lesions, or uterine scars (7). The presence of at least one direct TVUS feature was concluded to be strongly suggestive of adenomyosis, whereas the presence of indirect features with no direct features was agreed to be diagnostically inconclusive (7).

There is limited research into the correlation of clinical symptoms with proposed MUSA features. Some studies have shown a relationship between increased number of TVUS features and dysmenorrhea, heavy menstrual bleeding, and infertility (8–10). There is a small amount of mixed evidence regarding correlations between specific MUSA features (e.g. junctional zone involvement) and fertility-specific adenomyosis symptoms (11, 12). The overall impact of direct and indirect MUSA features on the clinical picture of adenomyosis is not well understood (7). The primary objective of this study was to assess the general prevalence of MUSA ultrasound criteria of adenomyosis, and the rates, location, and size of these ultrasound features in patients experiencing infertility, heavy menstrual bleeding and pelvic pain.

## Methods

We conducted an analytical cross-sectional study with patients presenting for TVUS at a single center imaging facility between May 2019 and August 2023 with institutional research ethics board approval. Inclusion criteria were individuals age 18 - 52, pre-menopausal, English-speaking, and undergoing a TVUS for any gynecologic indication. Individuals with previous hysterectomy and/or bilateral oophorectomy, menopause (defined as >1 year without menses), or active gynecologic malignancy were excluded. All eligible participants presenting to the imaging center were approached for recruitment on days where a research assistant was available.

Consenting patients completed a questionnaire designed to assess pelvic pain, heavy menstrual bleeding, and infertility (**Supplementary Figure 1**). Pelvic pain was evaluated by the presence of dyspareunia and assessment of dysmenorrhea using a numerical rating scale (NRS). Heavy menstrual bleeding was assessed using a validated menstrual bleeding questionnaire (MBQ) (13). Infertility was assessed by collecting data on difficulty conceiving for >1 year, adverse pregnancy outcomes, referral to fertility specialist, and use of assistive reproductive technologies. Other self-reported data collected included demographic information, gravidity and parity, known gynecologic diagnoses (e.g. fibroids, endometriosis, pelvic inflammatory disease (PID)), and use of any medications for dysmenorrhea.

Images were captured using the GE Voluson S8 ultrasound machine, with intracavitary probes that operate across a broad multi-frequency bandwidth of 3.0 MHz to 9.0 MHz. Two-dimensional imaging was performed for uterine assessment; coronal plan imaging (three-dimensional) was not routinely performed. Patients were scheduled for imaging early in the menstrual cycle. All sonographers who participated in gathering study data have American Registry for Diagnostic Medical Sonography (ARDMS) certification and are dedicated OB/GYN sonographers, part of a group of 30 clinics all specializing in OB/GYN imaging. All have received extensive training in using the MUSA criteria during medical education days from senior sonographers, radiologists and gynecologists specializing in the area, and followed a standardized TVUS protocol for patients participating in the study (5, 7). TVUS images were assessed by an expert radiologist (A.H.), who was blinded to subject survey data, using a standardized reporting form of MUSA criteria (**Supplementary Figure 2**) (5, 7).

Participants were divided into groups based on TVUS findings of adenomyosis (direct features, indirect features with no direct features, no findings). Chosen comparisons included dyspareunia (dichotomous), dysmenorrhea (continuous; range 0-10), self-reported infertility or fertility-related healthcare use (dichotomous; participants who answered ‘yes’ to experiencing infertility >1 year or seeing fertility specialist), and heavy bleeding measured by total MBQ (continuous; range 0–75 points). Secondary comparisons were made between increasing number of features, increasing size of lesions, increasing severity of adenomyosis, and location of adenomyosis lesion and these clinical outcomes.

Descriptive statistics were reported with median (IQR) for continuous variables and frequency (percentage) for categorical variables. Between-group differences in the four survey-reported symptoms outcomes and patient characteristics (e.g., age, BMI, gravida, parity, diagnosed fibroids, endometriosis, PID, medication use for dysmenorrhea) were assessed using Kruskal-Wallis, Mann-Whitney U, Pearson’s chi-squared, or Fisher’s exact tests, as appropriate. Summary statistics and group comparisons were also conducted for each type of MUSA feature using the same methods. Additionally, survey-reported symptoms were compared by uterine location, feature type (focal/diffuse/mixed), size of lesion, and layer of uterine involvement (inner/middle/outer myometrium) relative to those without such features (5). To adjust for multiple comparisons when there were five or more tests for a given variable, p-values were multiplied by the number of tests performed. Missing data were assumed to be missing at random and excluded.

Univariable regression models evaluated associations between symptoms and presence of direct/indirect features, number of features, and size of lesions with no features group as reference. Additional univariable models assessed the relationship with clinically relevant covariates and variables differing significantly between direct and indirect feature groups (**Table 1**). Multivariable models were adjusted for these variables as well as those with p<0.1 in univariable analysis or deemed clinically relevant, including comorbid conditions such as endometriosis and fibroids. Both endometriosis and fibroids were defined by self-reported diagnosis on survey or diagnostic findings on TVUS. Logistic regression was used for dichotomous outcomes and linear regression for normally distributed continuous outcomes. Based on the achieved sample sizes of 31 participants with direct features and 84 participants with no ultrasonographic features of adenomyosis, and an estimated standard deviation of approximately 10.3 points for the MBQ score, the study had 80% power, at a two-sided α of 0.05, to detect a between-group difference of approximately 6.1 points in total MBQ score. All statistical tests were two-sided; p-values <0.05 were considered significant. Analyses were conducted using R version 4.2.1.

**Table 1:** Demographic data.

Demographic variables		Overall	Direct TVUS features	Indirect TVUS features	No TVUS features	p-value
# participants		271	31	156	84	
Age (median [IQR])		36.0 [32.5, 39.0]	38.00 [33.5, 42.5]	37.0 [33.0, 40.0]	34.0 [30.0, 36.2]	<0.001
BMI (median [IQR])		24.0 [21.1, 29.0]	24.0 [22.7, 26.7]	24.1 [21.0, 29.0]	23.0 [21.1, 28.0]	0.545
Gravida (%)	0	128 (47.8)	11 (36.7)	70 (45.2)	47 (56.6)	Not available
	1	67 (25.0)	5 (16.7)	39 (25.2)	23 (27.7)	
	2	37 (13.8)	6 (20.0)	24 (15.5)	7 (8.4)	
	3	21 (7.8)	3 (10.0)	13 (8.4)	5 (6.0)	
	≥ 4	15 (5.5)	5 (16.1)	9 (5.8)	1 (1.2)	
Parity (%)	0	198 (74.2)	18 (60.0)	112 (72.7)	68 (81.9)	0.274
	1	37 (13.9)	5 (16.7)	23 (14.9)	9 (10.8)	
	2	25 (9.4)	5 (16.7)	15 (9.7)	5 (6.0)	
	3	6 (2.2)	2 (6.7)	3 (1.9)	1 (1.2)	
	4	0 (0.0)	0 (0.0)	0 (0.0)	0 (0.0)	
	5	1 (0.4)	0 (0.0)	1 (0.6)	0 (0.0)	
Fibroids diagnosis (%)		83 (30.6)	14 (45.2)	62 (39.7)	7 (8.3)	<0.001
Endometriosis diagnosis (%)		45 (16.6)	7 (22.6)	26 (16.7)	12 (14.3)	0.569
Any medication for dysmenorrhea		195 (73.3)	21 (70.0)	125 (80.6)	49 (60.5)	0.004
Hormonal medication for dysmenorrhea		45 (16.6)	7 (22.6)	26 (16.7)	12 (14.3)	0.569

## Results

A total of 271 subjects were recruited to our study based on inclusion and exclusion criteria met on the day of their appointment. Participants completed their scheduled ultrasound and were grouped into those with ≥1 direct features (n = 31, 11.4%), ≥1 indirect with no direct features (n = 156, 57.6%), and a control group with no features of adenomyosis on TVUS (n = 84, 31.0%) (**Table 1**). All 31 patients with direct features were also found to have at least one indirect feature present on ultrasonography. Subjects with any adenomyosis features were significantly older (median age of direct 38.00, indirect without direct 37.00, control 34.00; p = <0.001), more likely to have fibroids (45.2% of direct, 39.7% of indirect without direct, 8.3% of control; p = <0.001), and more likely to be taking medication for dysmenorrhea (70.0% of direct, 80.6% of indirect without direct, 60.5% of control; p = 0.004) (**Table 1**). Notably, there was no significant difference in prevalence of endometriosis (22.6% of direct, 16.7% of indirect without direct, 14.3% of control; p = 0.569) or use of hormone-based medications for dysmenorrhea (22.6% of direct, 16.7% of indirect without direct, 14.3% of control; p=0.569) between the three groups (**Table 1**).

In univariable analysis of TVUS features with clinical adenomyosis symptoms, with no feature group as reference (**Table 2**), direct features were associated with increased heavy bleeding MBQ score (β-coef 7.737 [95%CI 3.491-11.984], p < 0.001), whereas indirect (without direct) features were associated with infertility (OR 1.771 [95%CI 1.039, 3.041], p = 0.037) and dysmenorrhea (β-coef 0.777 [95%CI 0.065-1.488], p = 0.033). In multivariable analysis adjusted for potential confounders (**Table 2**), presence of direct features of adenomyosis on ultrasound resulted in a 5.955-point higher MBQ score for heavy bleeding (β-coef 5.955 [95%CI 1.970-9.939], p=0.004). When controlling for these covariates, indirect features were no longer significantly associated with infertility or dysmenorrhea.

**Table 2:** Univariable and multivariable regression exploring effect of direct and indirect TVUS features, increasing number of features, and increasing lesion size on clinical symptoms of adenomyosis compared to subject with no features.

Ultrasonographic findings	Infertility* (OR [95%CI], p-value)	Dyspareunia^†^ (OR [95%CI], p-value)	Dysmenorrhea^‡^ (β-coef [95%CI], p-value)	Heavy bleeding (MBQ Score)^‡^ (β-coef [95%CI], p-value)
Univariable analysis				
Direct features	1.619 [0.708-3.754], 0.255	1.902 [0.819-4.507], 0.137	1.030 [-0.076-2.136], 0.068	7.737 [3.491-11.984], <0.001
Indirect features	1.771 [1.039-3.041], 0.037	1.347 [0.784-2.330], 0.283	0.777 [0.065-1.488], 0.033	1.269 [-1.471-4.009], 0.363
Increasing number of features	1.160 [0.994-1.343], 0.059	1.127 [0.964-1.318], 0.132	0.296 [0.093-0.499], 0.004	1.204 [0.414-1.995], 0.003
Increasing lesion size	1.132 [0.694-1.848], 0.619	1.083 [0.671-1.747], 0.745	0.599 [-0.041-1.239], 0.066	5.291 [2.728-7.854], <0.001
Multivariable analysis				
Direct features	1.443 [0.582-3.576], 0.428	1.950 [0.773-4.917], 0.157	0.515 [-0.404-1.431], 0.270	5.955 [1.970-9.939], 0.004
Indirect features	1.652 [0.896-3.047], 0.108	1.337 [0.719-2.487], 0.359	0.110 [-0.513-0.734], 0.728	-0.760 [-3.487-1.966], 0.583
Increasing number of features	1.129 [0.944-1.350], 0.183	1.126 [0.939-1.349], 0.200	0.109 [-0.072-0.290], 0.236	0.669 (-0.135-1.475), 0.103
Increasing lesion size	1.084 [0.636-1.850], 0.766	1.008 [0.606-1.678], 0.974	0.404 [-0.147-0.955], 0.149	4.225 [1.537-6.912], 0.002

CI, confidence interval.

OR, odds ratio.

β-coef, regression coefficient.

*multivariable analysis adjusted for age, fibroids, endometriosis, prior pregnancy, use of medication for dysmenorrhea.

† multivariable analysis adjusted for age, fibroids, endometriosis, use of medication for dysmenorrhea, use of hormonal medication for dysmenorrhea.

‡ multivariable analysis adjusted for age, BMI, fibroids, endometriosis, use of medication for dysmenorrhea, use of hormonal medication for dysmenorrhea.

When assessing number of individual MUSA features (e.g. globular uterus) independently of direct and indirect groupings, there were 4 subjects (1.5%) with 1 feature of adenomyosis on TVUS, 14 (5.2%) with 2 features, and 169 (62.4%) with 3 or more features. In univariate analysis (**Table 2**), each additional MUSA feature was associated with a significant increase in dysmenorrhea scores (β-coef 0.296 [95% CI 0.093-0.499], p = 0.004) and MBQ score for heavy bleeding (β-coef 1.204 [95%CI 0.414-1.995], p = 0.003). In multivariable analysis, increasing number of TVUS features no longer exhibited statistically significant relationships with any clinical symptoms of adenomyosis.

In univariable models that examined the relationship between TVUS lesion size and clinical symptoms of adenomyosis, there was an association between largest diffuse lesion size and MBQ score for heavy bleeding (β-coef 5.291 [95% CI 2.728-7.854], p < 0.001) (**Table 2**). When controlling for age, BMI, fibroids, endometriosis, and any medication use in multivariable analysis, for every 1cm increase in diffuse lesion size, there was a respective 4.225-point increase in total MBQ score for heavy bleeding (95% CI [1.537-6.912], p=0.002). Examination of focal lesion size on univariable analysis revealed no relationship to clinical symptoms of adenomyosis.

There were 189 subjects (69.7%) with junctional zone (JZ) adenomyosis invasion, 171 (63.1%) with middle myometrium invasion, and 54 (19.9%) with outer myometrium invasion. Subjects with JZ invasion showed significantly higher dysmenorrhea scores (p=0.033) (**Table 3**). Those with middle myometrium invasion reported significantly increased rates of infertility compared to those without middle myometrial lesions (p = 0.006) (**Table 3**). Outer myometrial involvement was related with significantly higher heavy bleeding total MBQ score compared to those without outer layer involvement (p=0.002) (**Table 3**). All subjects with middle myometrial invasion had JZ invasion, and all but one subject with outer myometrial invasion had middle and JZ invasion. There was no significant association with anatomic location of adenomyosis TVUS findings (anterior, posterior, lateral, fundal) and pelvic pain, heavy bleeding, and infertility outcomes.

**Table 3:** Location of adenomyosis lesions on TVUS and the effect on clinical symptoms of adenomyosis compared to subject with no features at that location.

Disease location	Infertility (n, (%), p-value*)	Dyspareunia (n, (%), p-value*)	Dysmenorrhea (median [IQR], p-value*)	Heavy bleeding (MBQ score) (median [IQR], p-value*)
Junctional zone/Inner myometrium	106 (56.1), 0.087	92 (48.9), 0.349	5.25 [3.00, 7.00], 0.033	15.00 [9.50, 23.00], 0.121
Middle myometrium	101 (59.1), 0.006	82 (48.2), 0.626	5.00 [3.00, 7.00], 0.121	15.00 [10.00, 23.75], 0.087
Outer myometrium	30 (55.6), 0.714	27 (50.9), 0.604	6.00 [4.00, 8.00], 0.054	20.00 [11.00, 28.25], 0.001

*p-values derived from comparing junctional zone/middle myometrium/outer myometrium groups to those with no involvement at that given location. IQR, interquartile range.

## Discussion

Our study found that approximately 1 in 10 (11.4%) subjects had direct features of adenomyosis on TVUS, considered strongly suggestive of adenomyosis diagnosis based on the definition from Harmsen et al.’s consensus statement (7). This prevalence of adenomyosis is consistent with other literature in both the general and subfertility populations (2, 14). Moreover, all subjects with direct features had at least one or more concomitant indirect features on ultrasound. This is in keeping with Harmsen et al.’s consensus that indirect features will present as secondary sign when there is evidence of ectopic endometrial tissue (7).

### Direct and indirect features

Our data indicates that presence of direct features of adenomyosis on TVUS is associated with increased heavy bleeding scores, independent of confounders. Other studies have found diffuse adenomyosis is associated with heavy bleeding and demonstrated a positive correlation between increasing number of TVUS features and menstrual blood loss (9, 15), however, to our knowledge, no prior study has asserted a relationship between type of MUSA features and heavy bleeding. Characterizing this relationship may encourage radiologists to comment on the specific presence of direct features in ultrasound reports, thus guiding clinicians to initiate targeted medical therapy due to increased diagnostic certainty that adenomyosis is contributing to patient symptoms. While presence of solely indirect features initially showed relationships to infertility and dysmenorrhea on univariate analysis, this association was no longer significant when controlling for potential confounders such as fibroids and endometriosis. It is possible that this loss of significance on multivariable analysis is due to true confounding from presence of fibroids or endometriosis, or that there remains an independent association between indirect features and adenomyosis too subtle to be detected by the sample size of this study. Until further research with larger sample size can be completed, this observation remains in keeping with the suspicion that presence of indirect features can be a result of other uterine pathologies with similar symptomatology (7). These observations support Harmsen et al.’s consensus that presence of indirect features alone should not be used to determine conclusive adenomyosis diagnosis, but rather should prompt further investigation (7).

### Layer of uterine involvement

Similarly, in addition to identifying direct and indirect features, Van den Bosch et al. propose dedicated assessment of adenomyosis uterine layer involvement within the junctional zone/inner myometrium, middle myometrium, and outer myometrium (5). Middle myometrium lesions demonstrated significantly higher rates of self-reported infertility or fertility-related healthcare use (**Table 3**). We hypothesize that this disease pattern may elicit the local inflammatory changes and hyperperistalsis thought to be the mechanism of infertility in adenomyosis (4, 16). Outer myometrial involvement was related to heavy bleeding. We suspect this disease pattern may increase bleeding due to presence of vascular adenomyotic tissue in the outer myometrium, the major contractile tissue of the uterus (17). While there was a significant association between JZ/inner myometrial disease and dysmenorrhea, we interpret this relationship with caution as JZ irregularities were assessed as an indirect feature of adenomyosis, which showed no relationship to dysmenorrhea on multivariable analysis. Furthermore, when classifying layer involvement, four subjects were identified as having JZ/inner myometrial involvement who were not identified as having an irregular or interrupted junctional zone upon initial assessment of MUSA criteria. These subjects all demonstrated extensive multilayer disease involving the middle, and frequently outer, myometrium. We suspect that for rarer cases of individuals with extensive disease, it becomes challenging to differentiate between uterine muscle layers, particularly when assessing the junctional zone. This is in keeping with the challenges with intra- and inter-rater reliability when assessing for interrupted or irregular junctional zone reported in the literature (7, 19). While detection of middle and outer myometrial disease involvement may have clinical utility in relation to adenomyosis symptoms, further investigation should be done to direct reporting of layer of involvement for individuals with extensive disease or comorbid myometrial pathologies (e.g. fibroids), and this reporting should be interpreted cautiously for this subset of patients.

### Size of lesions

Previous literature suggests that presence and size of diffuse lesions are correlated with increased heavy bleeding scores (9, 18). Our data supports these findings, demonstrating that for every 1cm increase in diffuse lesion size, heavy bleeding MBQ scores increase by 4 points. As such, when diffuse lesions are present, it may be reasonable for diagnosticians to report size of largest lesion to help guide clinician decision-making for treatment of heavy menstrual bleeding.

### Increasing lesion number

The results of our study did not display a convincing association between increasing number of MUSA features and clinical symptoms after controlled for confounding variables. In previous literature with positive signals, associations were not controlled for confounding (8, 9), or had a small coefficient of association once covariates were eliminated (10). Without further research, it is difficult to determine the importance of considering number of features when evaluating expected symptom severity.

### Strengths and limitations

To our knowledge, this is the first study to examine the relationship between MUSA features all three key symptoms of the adenomyosis: infertility, pelvic pain, and heavy menstrual bleeding. By recruiting from a single center with images interpreted by a sole radiologist, there was consistency and standardization in data collection. By recruiting participants receiving imaging for any indication, our findings are most applicable to the general gynecologic patient population. While we attempted to include a full scope of adenomyosis symptoms in our analysis, adenomyosis is known to affect other FIGO abnormal uterine bleeding system 1 parameters beyond heavy bleeding, such as regularity and frequency. As the MBQ was not validated to capture these symptoms, further investigation is warranted to determine whether there is association between MUSA features and bleeding patterns such as regularity and frequency. Similarly, we were limited in our ability to analyse infertility due to lack of validated tools available, and as a result, our definition of infertility includes patients who have had difficulty conceiving for >1 year as well as patient who have had care from a fertility specialist. These parameters could have led to the inclusion of a small number of patients who sought specialist care for other indications such as fertility preservation or genetic disease prevention, thus all infertility related conclusions should be interpreted with this in mind. Finally, our main limitation was participant recruitment due to resource constraints from a single-center study and restrictions in research and clinical practice posed by the COVID-19 pandemic during our study period. To account for limited sample size and reduce Type 1 error, we grouped TVUS features into direct and indirect rather than analyzing individual MUSA features, grouped survey outcomes into four categories of adenomyosis symptoms, and used the Bonferroni correction to adjust p-values to account for multiple comparisons when appropriate. Due to the study’s comprehensive assessment of adenomyosis symptoms and TVUS features using available clinical tools, our effort to adjust for limitations in sample size and multiple comparisons, and reassuring power calculations, we believe our findings have the potential to contribute to the validation of the MUSA consensus statements, guiding the criteria with which clinicians diagnose adenomyosis on ultrasound (5, 7).

## Conclusions

When examining the association between clinical symptoms of adenomyosis and MUSA features on TVUS, we found presence of direct features and increasing diffuse lesion size to be significantly associated with heavier menstrual bleeding, even when controlling for potential confounders such as endometriosis and fibroids. There remains no individual MUSA feature that was associated to adenomyosis-related pelvic pain and infertility in the general gynecologic population, however layer of uterine involvement seem to demonstrate a relationship with these symptoms.

## Data Availability

The raw data supporting the conclusions of this article will be made available by the authors, without undue reservation.

## References

[1] García-Solares J Donnez J Donnez O Dolmans MM . Pathogenesis of uterine adenomyosis: invagination or metaplasia? Fertil Steril. (2018) 109:371–9. doi: 10.1016/j.fertnstert.2017.12.030 29566849

[2] Mishra I Melo P Easter C Sephton V Dhillon-Smith R Coomarasamy A . Prevalence of adenomyosis in women with subfertility: systematic review and meta-analysis. Ultrasound Obstet Gynecol. (2023) 62:23–41. doi: 10.1002/uog.26159 36647238

[3] Puente JM Fabris A Patel J Patel A Cerrillo M Requena A . Adenomyosis in infertile women: Prevalence and the role of 3D ultrasound as a marker of severity of the disease. Reprod Biol Endocrinol. (2016) 14:1–9. doi: 10.1186/s12958-016-0185-6 27645154 PMC5029059

[4] Harada T Khine YM Kaponis A Nikellis T Decavalas G Taniguchi F . The impact of adenomyosis on women’s fertility. Obstet Gynecol Surv. (2016) 71:557–68. doi: 10.1097/OGX.0000000000000346 27640610 PMC5049976

[5] Van den Bosch T de Bruijn AM de Leeuw RA Dueholm M Exacoustos C Valentin L . Sonographic classification and reporting system for diagnosing adenomyosis. Ultrasound Obstet Gynecol. (2018) 53:576–82. doi: 10.1002/uog.19096 29790217

[6] Dason ES Maxim M Sanders A Papillon-Smith J Ng D Chan C . Guideline No. 437: Diagnosis and management of adenomyosis. J Obstet Gynaecol Can. (2023) 45:417–29. doi: 10.1016/j.jogc.2023.04.008 37244746

[7] Harmsen MJ Van den Bosch T de Leeuw RA Dueholm M Exacoustos C Valentin L . Consensus on revised definitions of Morphological Uterus Sonographic Assessment (MUSA) features of adenomyosis: results of modified Delphi procedure. Ultrasound Obstet Gynecol. (2022) 60:118–31. doi: 10.1002/uog.24786 34587658 PMC9328356

[8] Decter D Arbib N Markovitz H Seidman DS Eisenberg VH . Sonographic signs of adenomyosis in women with endometriosis are associated with infertility. J Clin Med. (2021) 10:2355. doi: 10.3390/jcm10112355 34072021 PMC8198130

[9] Pinzauti S Lazzeri L Tosti C Centini G Orlandini C Luisi S . Transvaginal sonographic features of diffuse adenomyosis in 18–30-year-old nulligravid women without endometriosis: association with symptoms. Ultrasound Obstet Gynecol. (2015) 46:730–6. doi: 10.1002/uog.14834 25728241

[10] Naftalin J Hoo W Nunes N Holland T Mavrelos D Jurkovic D . Association between ultrasound features of adenomyosis and severity of menstrual pain. Ultrasound Obstet Gynecol. (2016) 47:779–83. doi: 10.1002/uog.15798 26499878

[11] Dason ES Maxim M Hartman A Li Q Kanji S Li T . Pregnancy outcomes with donor oocyte embryos in patients diagnosed with adenomyosis using the Morphological Uterus Sonographic Assessment criteria. Fertil Steril. (2023) 119:484–9. doi: 10.1016/j.fertnstert.2022.12.021 36539054

[12] Brandt NB Beneduce G Hartwig TS Vexø LE Madsen EP Dahlberg ES . O-218 Symptoms and signs of adenomyosis according to the revised MUSA criteria in 375 women with pregnancy loss and known ploidy of the pregnancy loss. Hum Reprod. (2025) 40. doi: 10.1093/humrep/deaf097.218

[13] Matteson KA Scott DM Raker CA Clark MA . The menstrual bleeding questionnaire: development and validation of a comprehensive patient-reported outcome instrument for heavy menstrual bleeding. BJOG. (2015) 122:681–9. doi: 10.1111/1471-0528.13273 25615842 PMC4373964

[14] Orlov S Jokubkiene L . Prevalence of endometriosis and adenomyosis at transvaginal ultrasound examination in symptomatic women. Acta Obstet Gynecol Scand. (2022) 101:524–31. doi: 10.1111/aogs.14337 35224723 PMC9564812

[15] Naftalin J Hoo W Pateman K Mavrelos D Foo X Jurkovic D . Is adenomyosis associated with menorrhagia? Hum Reprod. (2014) 29:473–9. doi: 10.1093/humrep/det451 24408315

[16] Vercellini P Viganò P Bandini V Buggio L Berlanda N Somigliana E . Association of endometriosis and adenomyosis with pregnancy and infertility. Fertil Steril. (2023) 119:727–40. doi: 10.1016/j.fertnstert.2023.03.018 36948440

[17] Zhai J Vannuccini S Petraglia F Giudice LC . Adenomyosis: mechanisms and pathogenesis. Semin Reprod Med. (2020) 38:129. doi: 10.1055/s-0040-1716687 33032339 PMC7932680

[18] Exacoustos C Morosetti G Conway F Camilli S Martire FG Lazzeri L . New sonographic classification of adenomyosis: Do type and degree of adenomyosis correlate to severity of symptoms? J Minim Invasive Gynecol. (2020) 27:1308–15. doi: 10.1016/j.jmig.2019.09.788 31600574

[19] Rasmussen CK Hansen ES Dueholm M . Inter‐rater agreement in the diagnosis of adenomyosis by 2‐ and 3‐dimensional transvaginal ultrasonography. J Ultrasound Med. (2019) 38:657–66. doi: 10.1002/jum.14735 30182497

