# Supplementary material for: The relationship between symptoms of adenomyosis and current ultrasonographic diagnostic criteria
Source: Front Endocrinol (Lausanne). 2026 Oct 7;17:1924781. doi: 10.3389/fendo.2026.1924781 (PMC13683137; doi:10.3389/fendo.2026.1924781)
Supplement: Supplementary file 1 [file Supplementaryfile1.docx]

**Supplemental Figure 1: Patient Survey**

**ACCESSION NUMBER :**

**Project Title:** A prospective observational study assessing prevalence of transvaginal ultrasound features of adenomyosis in a general gynecologic population

**Please answer the following questions to the best of your ability :**

1. Have you ever had a surgical removal of your uterus (hysterectomy) or of your ovaries (oophorectomy)? Please choose one :

- Yes
- No

2. Do you current have, or have you ever had, a cancer of your uterus or ovaries? Please choose one :

- Yes
- No

3. What is your age? Please write your answer on the line :

________________________________________________________________

4. How tall are you? Please write your answer on the line :

________________________________________________________________

5. What is your current weight? Please write your answer on the line :

________________________________________________________________

6. What is your Ethnicity? Please Choose one:

- Asian
- Black/African American
- White/Caucasian
- Aboriginal
- Latin American
- Prefer not to say
- Other, Please Describe: _________________________________________________

7. What was the first day of your last period (approximately)? Please write your answer on the line :

________________________________________________________________

8. Why are you having an ultrasound today? Please choose all that apply:

- Painful periods
- Heavy periods
- Irregular periods
- Pelvic pain
- Infertility
- Painful sex
- Other: please describe: _____________________________________________________________

9. Have you been diagnosed with any of the following Gynaecological diagnoses? Please choose all that apply:

- Uterine Fibroids
- Endometriosis
- Pelvic Inflammatory Disease
- Adenomyosis
- Endometrial Polyp
- Endometrial Cancer
- Ovarian Cancer
- Ovarian Cysts
- Infertility
- Miscarriage
- Polycystic Ovarian Syndrome (PCOS)
- Other, please describe: ______________________________________________________________

10. If you **have endometriosis**, how was this confirmed? Please choose all that apply

- Your doctor told you
- Ultrasound/MRI
- Surgery
- Other, please describe: ______________________________________________________________

11. Have you ever experienced pain with sex? Please choose one:
    - Yes
    - No
12. Please use following scale to indicate how painful your periods are. Please **mark an X** on the number that corresponds to your experience with pain during an average period: *0 – no pain, 5= moderate pain, 10=worst possible pain*

13. When do you typically experience pain during your menstrual cycle? Please choose one :
    - Before my period comes
    - During my period
    - Before and during my period
    - After my period ends
    - Before, during and after my period
    - I always have pain
    - I do not experience pain with my periods
14. Have you ever stayed home from work or school because of your painful periods? Please choose one:

- Yes
- No

15. Have you ever seen a doctor because of how painful your periods are? Please choose one:

- Yes
- No

16. What medication have you used to help with your pain during your periods in the last 6 months? Please choose all that apply:

- Acetaminophen (Tylenol)
- Anti-inflammatory medications (Advil, Ibuprofen, Aleve, Naproxen, Motrin)
- Narcotics (Tylenol #3, Codeine, Morphine, Oxycodone, Hydromorphone, Dilaudid)
- Other, please describe:
- I have not used any medication(s)

17. Has your gynecologist or family doctor prescribed any of the following medications for your painful periods OR your heavy bleeding in the last 6 months? Please choose all that apply:

- Anti-inflammatory medications (Advil, Ibuprofen, Aleve, Naproxen)
- Tranexamic Acid (Cyclokapron)
- Birth Control Pills
- Fibristal
- Lupron injections
- Mirena intrauterine device
- Depo-Provera
- Visanne or other oral progesterone
- Yes, but I don’t remember the name of the medication
- Other, please describe: ______________________________________________________________
- I have not been prescribed any medication(s)

18. Have you ever been pregnant? Please choose one:
    - Yes
    - No
19. If yes to the previous question, how many times have you been pregnant (including miscarriage, abortion and ectopic pregnancies)? Please choose one:
    - 1
    - 2
    - 3
    - 4
    - 5
    - More than 5, please indicate number:
20. How many children have you given birth to?
    - 0
    - 1
    - 2
    - 3
    - 4
    - 5
    - more than 5, please indicate number:
21. Have you ever had a miscarriage? Please choose one:
    - Yes, please indicate how many miscarriages you have had #____
    - No
22. Have you ever had an ectopic pregnancy? Please choose one:
    - Yes, please indicate how many ectopic pregnancies you have had #__

- No

23. Have you ever had infertility (difficulty getting pregnant) for more than 6 months? Please choose one:
    - Yes
    - No
24. Have you ever had infertility (difficulty getting pregnant) for more than one year? Please choose one:
    - Yes
    - No
25. Have you ever seen or are you currently seeing an infertility specialist?

- Yes
- No

26. Have you ever or are you currently undergoing fertility treatment (fertility drugs, intrauterine insemination or in vitro fertilization)?

- Yes
- No
