# Supplementary material for: The relationship between symptoms of adenomyosis and current ultrasonographic diagnostic criteria
Source: Front Endocrinol (Lausanne). 2026 Oct 7;17:1924781. doi: 10.3389/fendo.2026.1924781 (PMC13683137; doi:10.3389/fendo.2026.1924781)
Supplement: Supplementary file 2 [file Supplementaryfile2.docx]

**Supplemental Figure 2: Radiology Report Form**

**Patient Study Code: ____________________________________________________________**

**Anterior-Posterior diameter of uterine cavity : _____________________________________**

**Size of Uterus: ________________________________________________________________**

**Features of Adenomyosis :**

| **Features** | **Check if feature present** | | |
| --- | --- | --- | --- |
| **Globular enlarged uterus** |  | | |
| **Adenomyoma : ill-defined nodular heterogenous myometrial mass hard to correctly measure** |  | | **Size :** |
| **Fan shaped shadowing** |  | | |
| **Asymmetrical thickening of the myometrium** |  | | |
| **Echogenic subendometrial lines and buds** |  | | |
| **Myometrial cysts** |  | Largest Cyst Diameter: | Presence of echogenic ring *(checkmark)*: |
|  |  | More than one *(checkmark)*: |  |
| **Translesional vascularity** |  | | |
| **Hyperechoic echogenic islands** |  | | |
| **Irregular or interrupted junctional zone** |  | | |

**Disease Location :**

*Checkmark may be placed in both “focal” and diffuse” categories*

| **Location** | **Check if Focal (more than 25% of lesion is surrounded by normal myometrium)** | **Check if Diffuse (total involvement of myometrium exceeds 25% of uterine corpus)** |
| --- | --- | --- |
| **Anterior** |  |  |
| **Posterior** |  |  |
| **Lateral right** |  |  |
| **Lateral left** |  |  |
| **Fundal** |  |  |

**Please indicate the lesion size :**

*If a check mark has been placed in one or both of the categories above, please indicate the lesion size below.*

**Focal lesion (largest diameter) :**

**Diffuse lesion (thickness of myometrial wall):**

**Myometrial Layer Involvement :**

| **Location** | **Check if Present** |
| --- | --- |
| **Junctional Zone (inner myometrium/subendometrial layer)** |  |
| **Middle myometrium** |  |
| **Outer myometrium (subserosal)** |  |

**Extent of Disease :**

*Please place checkmark in only one category*

| **Extent** | **Check one option** |
| --- | --- |
| **Mild (<25% of uterine corpus)** |  |
| **Moderate (25-50% of uterine corpus)** |  |
| **Severe (>50% of uterine corpus)** |  |

**Please indicate if any of the following pathology was also present :**

| **Pathology** | **Check if Pathology was present** |
| --- | --- |
| **Fibroids** |  |
| **Polyps** |  |
| **Endometriosis** |  |
| **Endometrial mass suggestive of cancer** |  |
| **Other :** |  |
